# Integrative single-nucleus transcriptomic analysis identifies UBE2C+ proliferative tumor cells and immune-stromal regulatory crosstalk in small cell lung cancer

**DOI:** 10.3389/fimmu.2026.1902738

**Published:** 2026-08-04

**Authors:** Hongling Jia, Yongxuan An, Bing Chen, Yuqi Zhang, Yifei Xu, Feilong Guan, Qian Wu, Lili Sun, Yajing Li, Junjie Bi, Xiuli Ji, Zhanjun Qiu

**Affiliations:** 1 College of First Clinical Medicine, Shandong University of Traditional Chinese Medicine, Jinan, China; 2 Department of Plastic and Reconstructive Surgery, Shanghai Ninth People’s Hospital, School of Medicine, Shanghai Jiao Tong University, Shanghai, China; 3 Department of Thoracic Surgery, Anhui Chest Hospital, Hefei, Anhui, China; 4 Department of Respiratory and Critical Care Medicine, Anhui Public Health Clinical Center, Hefei, Anhui, China; 5 Department of Gerontology, The Affiliated Hospital of Shandong University of Traditional Chinese Medicine, Jinan, China; 6 Guang’anmen Hospital Jinan Hospital, China Academy of Chinese Medical Sciences, Jinan, China

**Keywords:** cancer-immunity regulome, cell-cell communication, immune-stromal crosstalk, single-nucleus RNA sequencing, small cell lung cancer, UBE2C

## Abstract

**Background:**

Small cell lung cancer (SCLC) is an aggressive neuroendocrine malignancy characterized by rapid proliferation, early dissemination, and limited durable benefit from current chemoimmunotherapy. Although immune checkpoint blockade has modestly improved clinical outcomes, the regulatory logic linking malignant cell states to the tumor immune microenvironment remains incompletely understood. Here, we applied an integrative single-nucleus transcriptomic framework to dissect tumor cell heterogeneity, regulatory programs, and immune-stromal communication networks in SCLC.

**Methods:**

Publicly available Single-nucleus RNA sequencing data from primary and metastatic SCLC samples were analyzed using Seurat-based clustering, inferCNV-based malignant cell identification, differential expression analysis, pathway enrichment, metabolic and stemness scoring, pseudotime trajectory reconstruction, CellChat-mediated cell-cell communication inference, and transcription factor regulatory module analysis. A *UBE2C*-enriched proliferative tumor cell subpopulation was prioritized for functional validation. siRNA-mediated *UBE2C* knockdown was performed in DMS114 and NCI-H446 SCLC cell lines, followed by qRT-PCR, CCK-8, colony formation, transwell migration, and Annexin V/PI apoptosis assays.

**Results:**

Using snRNA-seq, we identified multiple cell types and resolved a *UBE2C*+ subpopulation with marked proliferative features. *UBE2C*+ subpopulation displayed strong G2/M-phase enrichment, elevated mitotic and cell cycle programs. And pseudotime analysis positioned C3 *UBE2C*+ tumor cells at a proliferative state during tumor cell state evolution. Cell-cell communication analysis suggested that this subpopulation might interact with macrophages and fibroblasts through GRN-SORT1 and THBS1-CD47/CD36 signaling axes, indicating a potential link between proliferative tumor states and candidate communication axes. Transcription factor module analysis further revealed enrichment of cell cycle-associated regulators, including MYBL2, NFYB, E2F2, TGIF1, and RXRG, in the C3 subpopulation. Functionally, *UBE2C* knockdown significantly suppressed proliferation, clonogenic growth, and migration while increasing apoptosis in SCLC cells.

**Conclusions:**

This study identified *UBE2C*+ proliferative tumor cells as a functionally relevant malignant subpopulation in SCLC and links this state to immune-stromal communication networks within the tumor microenvironment. By integrating single-nucleus transcriptomics, regulatory network inference, intercellular communication analysis, and *in vitro* validation, our findings nominate *UBE2C* as a potential candidate functional regulator and provide a systems-level framework for investigating the cancer-immunity regulome in SCLC.

## 1 Introduction

Currently, lung cancer remains a significant global health issue, being one of the malignant tumors with the highest incidence and mortality rates worldwide. Among these, lung cancer ranks second in terms of new cancer cases for both men and women, and first in terms of mortality rates. Lung cancer is mainly divided into two histological types: small cell lung cancer (SCLC) and non-SCLC. SCLC belongs to the category of neuroendocrine tumors, accounting for 13% to 15% of all newly diagnosed lung cancer patients. It is characterized by rapid growth, high invasiveness, early metastasis, and poor prognosis (1). Smoking is a major risk factor for SCLC, and most patients with SCLC have a history of heavy smoking. The incidence in non-smoking populations may be associated with occupational exposure, such as asbestos or polycyclic aromatic hydrocarbons, or with chronic obstructive pulmonary disease. Approximately 80% to 85% of patients are diagnosed with extensive-stage disease at the time of their initial diagnosis, making early detection of SCLC crucial, which limits the possibility of curative treatment (2).

With the advancement of science and technology, medical technology has also seen development, and the treatment strategies for SCLC are continuously progressing and updating. It is known that SCLC is highly sensitive to chemotherapy and radiotherapy, and these are important treatment strategies for SCLC. Although first-line etoposide plus platinum chemotherapy achieves an overall response rate of >50% (3), the recurrence of SCLC has consistently posed a significant challenge in clinical treatment. Most SCLC patients will relapse within a short period and progress to a drug-resistant state, and their subsequent treatment options and efficacy are limited (4). In parallel, immune checkpoint inhibitors (such as atezolizumab and durvalumab) combined with chemotherapy have been shown to extend overall survival in extensive stage SCLC, but the overall survival benefit is quite limited (5). Recently, with the progress of immune checkpoint inhibitors in extensive stage SCLC, immunotherapy for limited-stage SCLC has also become a research hotspot. However, there are still many clinical issues and a lack of high-level evidence to guide it (5). In terms of targeted therapy, the overall clinical translation effect of various drugs targeting DLL3, PARP, BCL-2, and other targets is not satisfactory (6). Overall, SCLC currently still faces multiple challenges, including a lack of treatment options, unclear resistance mechanisms, a shortage of effective targeted drugs, and reliable biomarkers.

Single-nucleus RNA sequencing (snRNA-seq) has revolutionized the study of cellular diversity. This technology can precisely characterize different cell states and their interaction networks, thereby providing a deeper understanding of cellular behavior in both healthy and diseased states. Traditional bulk-level transcriptome analysis often masks the contributions of low-abundance key cell populations and the heterogeneity of cell groups, whereas snRNA-seq provides a new tool for high-resolution analysis of gene expression patterns (7). By systematically characterizing the transcriptome maps of tumor cells (TCs), immune cells, and vascular endothelial cells at single-cell resolution, researchers can more accurately identify disease-specific cell subpopulations, thereby providing important insights into the key molecular mechanisms underlying the development of SCLC. By applying snRNA-seq technology, it helps to deeply analyze the complex intercellular communication networks within the microenvironment of SCLC (8). Using single-cell multi-omics technologies to identify new subpopulation-specific markers and immune checkpoint characteristics, combined with *in vitro* cell experiments for key gene validation, to discover new targets and markers, providing a basis for personalized treatment.

SCLC is a typical malignant tumor of epithelial cell origin, retaining epithelial characteristics in tissue development, immune phenotype, genetic lineage, and clinical behavior (9). Despite accumulating single-cell studies in lung cancer, the regulatory connection between proliferative malignant cell states and the immune-stromal microenvironment in SCLC remains insufficiently defined. In particular, it is unclear whether specific tumor cell subpopulations act as regulatory nodes that coordinate cell cycle progression, metabolic adaptation, transcription factor (TF) activity, and immune-stromal crosstalk. Addressing this question requires an integrative framework that moves beyond descriptive cell annotation toward the reconstruction of tumor-intrinsic and microenvironmental regulatory circuits.

In this study, we analyzed snRNA-seq profiles from primary and metastatic SCLC samples to construct a single-nucleus atlas of the SCLC tumor immune microenvironment. The single-nucleus RNA sequencing dataset (GSE303152) analyzed in this study was originally generated and published in a recent Nature (2025) study investigating neuronal activity-dependent mechanisms in small cell lung cancer (10). We identified malignant tumor cells using inferCNV and further resolved 4 distinct tumor cell subpopulations. By integrating differential expression, pathway enrichment, metabolic and stemness scoring, pseudotime reconstruction, CellChat-based intercellular communication inference, and TF regulatory module analysis, we identified a *UBE2C*+ proliferative tumor cell subpopulation linked to GRN- and THBS-mediated immune-stromal communication. Finally, we performed *UBE2C* knockdown in SCLC cell lines to experimentally validate its role in proliferation, migration, clonogenic growth, and apoptosis. These findings provide a systems-level perspective on the cancer-immunity regulome of SCLC and nominate *UBE2C*+ TCs as a potential targetable malignant state.

SCLC is classically characterized by a strong neuroendocrine lineage program, typically defined by the expression of canonical markers such as *ASCL1, NEUROD1, CHGA*, and *SYP* (11). However, increasing evidence from single-cell and lineage plasticity studies has demonstrated that SCLC is not a strictly fixed neuroendocrine disease, but instead exhibits substantial intratumoral heterogeneity and dynamic lineage plasticity (12, 13). In particular, non-neuroendocrine or neuroendocrine-low tumor cell states have been reported, which may arise through transcriptional reprogramming under microenvironmental pressure or therapeutic selection (14). In this context, the EPC-like tumor cell population identified in this study represents a distinct transcriptional state within the broader malignant epithelial compartment. Rather than being defined by classical neuroendocrine marker expression, EPC-like cells are characterized by strong proliferative and cell cycle-associated programs, suggesting a shift toward a less differentiated and more mitotically active state. Importantly, this does not contradict the established neuroendocrine framework of SCLC, but rather reflects the known plasticity between neuroendocrine and non-neuroendocrine tumor cell states. Therefore, we interpret EPC-like tumor cells as a malignant proliferative state that may coexist with, and potentially transition from, classical neuroendocrine tumor populations. This perspective is consistent with recent studies demonstrating that SCLC cells can dynamically switch between NE-high and NE-low states during disease progression and treatment exposure (15). Based on this rationale, the present study further investigates the functional characteristics and microenvironmental interactions of this EPC-like proliferative population within the SCLC ecosystem.

## 2 Methods

### 2.1 Sources of single-cell nuclear transcriptome sequencing data

The snRNA-seq data of SCLC were obtained from the Gene Expression Omnibus (GEO) database (https://www.ncbi.nlm.nih.gov/geo/). The accession number for the sample is GSE303152. Since this study uses existing data from public databases, there is no need to obtain approval from the ethics committee. Data analysis was conducted using the R package Seurat, with the relevant methodology cited in the reference number.

### 2.2 Analysis of single-cell nuclear transcriptome sequencing data

The raw single-cell nuclear transcriptome sequencing data were preprocessed using the Seurat package (v 4.4.0) and potential doublets were identified using the DoubletFinder package (v 2.0.3). Based on the following quality control standards, qualified cells were selected: gene detection numbers less than 300 or more than 6,000; total transcript molecule counts (nCount_RNA) less than 500 or more than 100,000; cells with mitochondrial gene expression ratios higher than 25% were excluded. For each sample, the expression levels are first converted to their relative proportions within the cell, then multiplied by 10,000 for normalization, and finally, the natural logarithm was taken after adding 1 to achieve standardization (to avoid logarithmic calculations on zero values). Based on the normalized expression matrix, the top 2,000 highly variable genes were selected, followed by scaling and principal component analysis. Using the R package Harmony (v 1.2.0), the first 30 principal components were used as input to correct for batch effects between samples. Based on the expression matrix corrected by Harmony, calculate the k-nearest neighbors and construct a shared nearest neighbor graph. By optimizing the modularity function, the clustering algorithm is improved, ultimately identifying cell clusters. The resulting cell clusters are visualized as a two-dimensional map using the Uniform Manifold Approximation and Projection (UMAP) dimensionality reduction technique (16–18).

### 2.3 Methods for pathway enrichment analysis of differentially expressed genes

This study employed Gene Ontology (GO), Kyoto Encyclopedia of Genes and Genomes (KEGG), and Gene Set Enrichment Analysis (GSEA) to perform functional annotation and pathway enrichment analysis on differentially expressed genes (DEGs). First, the FindAllMarkers function in the Seurat package was used to identify DEGs. Subsequently, the ClusterProfiler package (v 4.8.2) was used to perform functional enrichment analysis on these DEGs to identify significantly enriched GO terms (adjusted *P-value* < 0.05). In addition, we used the GSEA tool to evaluate the statistical significance and consistency differences between groups in the predefined gene sets by ranking all DEGs based on their expression levels. Furthermore, based on the DEG expression profiles of each cell subpopulation, the GSEA method was applied to explore the significantly enriched biological pathways in each cell cluster (19–21).

### 2.4 Measurement of CNVs

This study used the inferCNV tool (v 1.16.0) to quantitatively analyze the level of copy number variation (CNV). By performing copy number karyotype analysis on aneuploid cells within SCLC tissues, the distinction between non-TCs and malignant tumor cells was achieved (22–25).

### 2.5 Lineage trajectory analysis

Monocle was a tool that was used to order single cells along developmental trajectories and was capable of displaying the progression of cells through different stages of differentiation or development. This method mapped cells to a low-dimensional space through dimensionality reduction and calculated pseudotime values, thereby revealing the gene expression patterns of cells during their dynamic changes along developmental trajectories (including branching points) (26–28).

Slingshot, on the other hand, focused on identifying cell lineage trees by analyzing cell clusters. This tool constructed branching structures to represent different cell fates and their lineage branches. Although it did not directly compute pseudotime, Slingshot could effectively identify and map branching patterns within clustered cell populations in high-dimensional space (17).

### 2.6 Metabolism analysis and AUCell score analysis

Based on single-cell transcriptome data, we used the AUCell algorithm to score the activity levels of key metabolic pathways in various cell types. The set of metabolism-related genes was derived from KEGG metabolic pathways in the MSigDB database. By comparing the distribution characteristics of pathway scores between different cell subpopulations, we identified metabolic pathways with significant differences. Subsequently, the mean pathway scores of all cells within the same subpopulation were calculated to quantify the overall activity level of specific metabolic pathways in each cell subpopulation (29–31).

AUCell was an analytical strategy used to identify cell populations with transcriptional activity of target gene sets. The algorithm took a predefined gene set as input and calculates an enrichment score for each cell that reflects the overall activity of that gene set. This study used the AUCell method to assess the stemness levels of various cell subpopulations (32–37).

### 2.7 Cell-cell communication analysis

This study used the CellChat R package (v 1.6.1) for intercellular communication analysis. CellChat included a comprehensive database of signal molecule interactions, integrating known structural composition information of ligand-receptor interactions. This tool was based on a mass action model to infer cell state-specific signaling communication events from given single-cell sequencing data, thereby predicting interactions between different cell types (38–40). To deeply explore the interaction networks between different cell subpopulations in SCLC patients and their ligand-receptor pairs, this study utilized various visualization functions (41–43).

### 2.8 Single-cell regulatory network inference and clustering analysis

To characterize subpopulation-specific transcriptional regulatory programs, we conducted SCENIC analysis using the Python-based SCENIC package (v. 1.3.1). Potential regulatory interactions between transcription factors (TFs) and their downstream target genes were initially predicted with GRNBoost. These candidate regulatory modules were further filtered by DNA motif enrichment analysis to determine likely direct TF-target associations. Finally, AUCell was used to estimate regulon activity at the single-cell level, and the top 5 TFs with the highest activity scores were selected for downstream analysis (7, 8).

### 2.9 Cell lines and culture conditions

Human SCLC cell lines DMS114 and NCI-H446 were used in this study. DMS114 cells were obtained from Procell Life Science & Technology Co., Ltd. (Wuhan, China; Cat# CL-0752), and NCI-H446 cells were obtained from Sunncell Biotechnology Co., Ltd. (Wuhan, China; Cat# SNL-393). Cell line authentication was confirmed by short tandem repeat (STR) profiling, and all cells were routinely tested and confirmed to be free of mycoplasma contamination using a PCR-based Mycoplasma Detection Kit.

DMS114 cells were cultured in Waymouth’s MB 752/1 medium supplemented with 10% fetal bovine serum and 1% penicillin–streptomycin. NCI-H446 cells were maintained in RPMI-1640 medium supplemented with 10% fetal bovine serum and 1% penicillin–streptomycin. Cells were cultured at 37 °C in a humidified incubator containing 5% CO_2_ and passaged every 2-3 days to maintain exponential growth (43–46).

### 2.10 siRNA-mediated knockdown of *UBE2C*

Two independent small interfering RNAs targeting human *UBE2C*, designated si-*UBE2C*-1 and si-*UBE2C*-2, together with a non-targeting control siRNA, were synthesized by GenePharma (Shanghai, China). DMS114 and NCI-H446 cells were transfected with siRNAs at a final concentration of 50 nM using Lipofectamine™ RNAiMAX Transfection Reagent according to the manufacturer’s protocol.

Briefly, cells were seeded into 6-well plates and transfected when they reached approximately 50-70% confluence. After 6 h of transfection, the medium was replaced with fresh complete culture medium. Cells were harvested 48 h after transfection for quantitative real-time PCR (qRT-PCR) validation of *UBE2C* knockdown efficiency and for subsequent functional assays.

The experimental groups were as follows: si-Ctrl, si-*UBE2C*-1, and si-*UBE2C*-2. The use of two independent *UBE2C*-targeting siRNAs was intended to reduce potential off-target effects and strengthen the specificity of the observed phenotypes. The siRNA sequences are provided in **Supplementary Table 1** (47–50).

### 2.11 RNA extraction and qRT-PCR

Total RNA was extracted 48 h after transfection using TRIzol™ Reagent according to the manufacturer’s protocol. RNA concentration and purity were determined using a NanoDrop 2000 spectrophotometer. Complementary DNA was synthesized using the PrimeScript™ RT Reagent Kit.

qRT-PCR was performed using SYBR Green Master Mix on a real-time PCR detection system. Relative *UBE2C* mRNA expression was calculated using the 2^-ΔΔCt method, with GAPDH used as the internal reference gene. Each reaction was performed in triplicate, and three independent biological replicates were analyzed. Primer sequences are listed in **Supplementary Table 1** (51–54).

### 2.12 Colony formation assay

Colony formation assays were performed to evaluate long-term clonogenic growth. At 24-48 h after transfection, DMS114 and NCI-H446 cells were seeded into 6-well plates at a density of 800-1,000 cells per well and cultured for 10-14 days in complete medium. The medium was replaced every 3 days.

Colonies were fixed with 4% paraformaldehyde for 20 min and stained with 0.1% crystal violet for 15 min at room temperature. Colonies containing at least 50 cells were counted under a light microscope. Colony numbers were quantified using ImageJ software (19).

### 2.13 Cell proliferation assay (CCK-8)

Cell proliferation was assessed using the Cell Counting Kit-8 assay. At 24 h after siRNA transfection, DMS114 and NCI-H446 cells were seeded into 96-well plates at an appropriate density, typically 2 × 10³ to 3 × 10³ cells per well, with 5 replicate wells per group (55).

At 0, 24, 48, 72, and 96 h after seeding, 10 μL of CCK-8 reagent was added to each well and incubated for 2 h at 37 °C. The optical density (OD) at 450 nm was measured using a microplate reader to evaluate time-dependent changes in cell proliferation (56, 57).

### 2.14 Transwell migration assay

Cell migration was evaluated using transwell chambers with 8 μm pore polycarbonate membranes. 48 h after transfection, DMS114 or NCI-H446 cells were resuspended in serum-free medium and seeded into the upper chambers. For DMS114 cells, 3 × 10^4^ to 5 × 10^4^ cells were used per chamber; for NCI-H446 cells, 2 × 10^4^ to 4 × 10^4^ cells were used per chamber. Complete medium containing 10% FBS was added to the lower chamber as a chemoattractant.

After incubation for 16–24 h at 37 °C, non-migrated cells on the upper surface of the membrane were gently removed with a cotton swab. Migrated cells on the lower surface were fixed with 4% paraformaldehyde and stained with 0.1% crystal violet. Images were captured under a light microscope, and migrated cells were counted in 5 randomly selected fields per membrane (54, 58).

### 2.15 Apoptosis analysis by flow cytometry

Apoptosis was assessed using an Annexin V-FITC/PI Apoptosis Detection Kit. 48 h after siRNA transfection, both floating and adherent cells were collected to avoid underestimation of apoptotic cells. Cells were washed twice with cold PBS, collected by centrifugation, and then resuspended in 1× binding buffer.

Cells were stained with Annexin V-FITC and propidium iodide for 15 min at room temperature in the dark. Samples were analyzed by flow cytometry. Early apoptotic cells were defined as Annexin V^+^/PI^-^, and late apoptotic cells were defined as Annexin V^+^/PI^+^. The total apoptosis rate was calculated as the sum of early and late apoptotic fractions (59, 60).

### 2.16 Statistical analysis

All experiments were performed with at least three independent biological replicates. Data are presented as mean ± SD. Statistical analyses were performed using GraphPad Prism software. Comparisons among three groups were analyzed using one-way ANOVA followed by Tukey’s multiple-comparison test. For CCK-8 time-course assays, two-way ANOVA followed by multiple-comparison testing was used. A two-sided *P* value < 0.05 was considered statistically significant (61, 62).

## 3 Results

### 3.1 Single-cell landscape of SCLC

**Figure 1:**
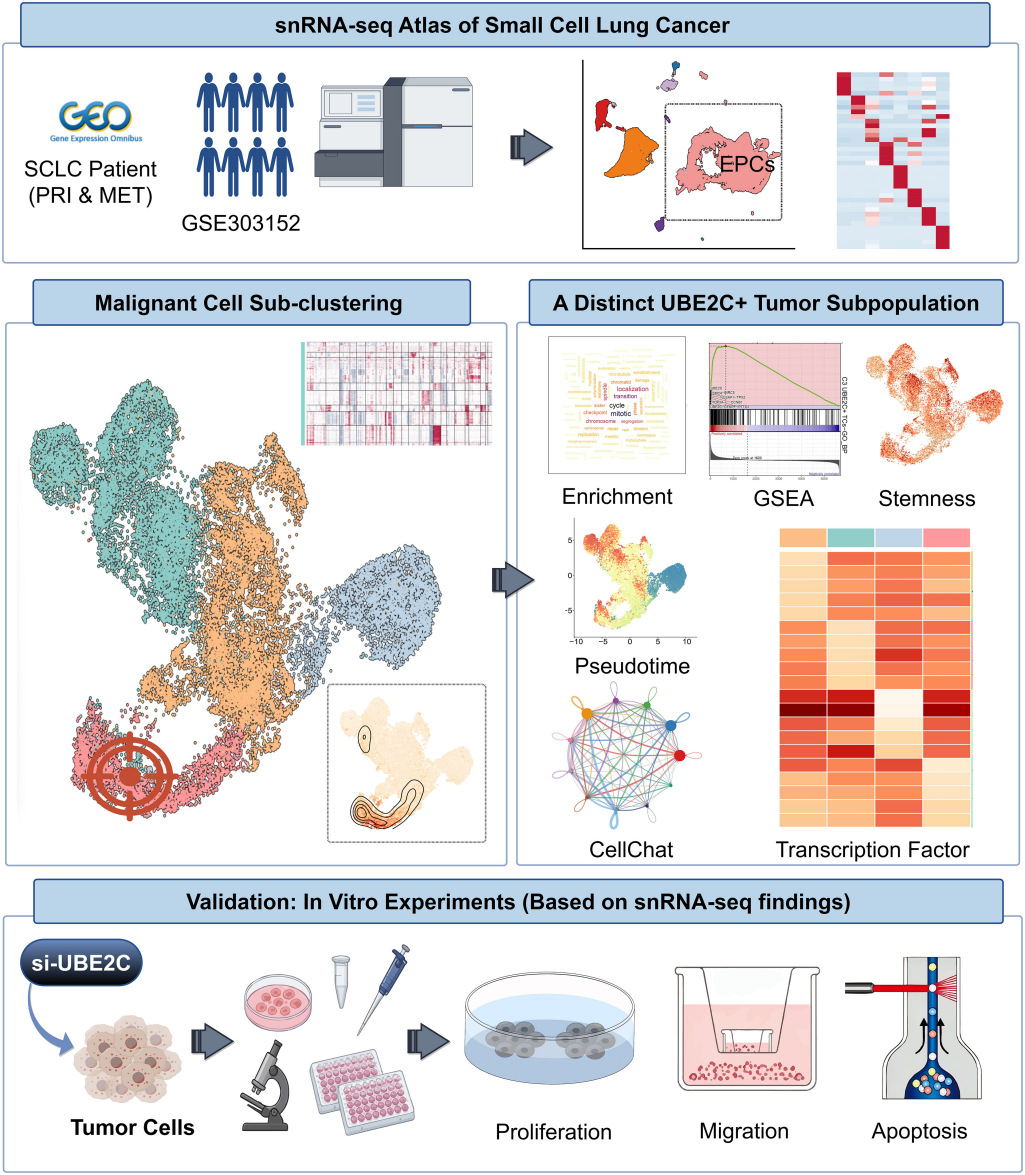
Workflow for constructing the snRNA-seq atlas of SCLC, identification and functional validation of *UBE2C*+ tumor cell subpopulations. Based on the dataset GSE303152 from the GEO database containing primary (PRI) and metastatic (MET) tumor samples of SCLC patients, after sequencing data processing, cell clustering, and selection of EPCs, malignant cell subpopulations were further analyzed to identify a unique tumor cell subpopulation characterized by high UBE2C expression. Subsequently, functional enrichment analysis, GSEA, stemness score, pseudotime analysis, cell communication analysis, and TF activity analysis were performed to systematically decipher the molecular features and potential biological functions of the *UBE2C*+ tumor subpopulation. Finally, based on the single-cell analysis results, the effect of *UBE2C* on malignant phenotypes such as proliferation, migration, and apoptosis of tumor cells was evaluated through *in vitro* si-*UBE2C* interference experiments.

**Figure 2:**
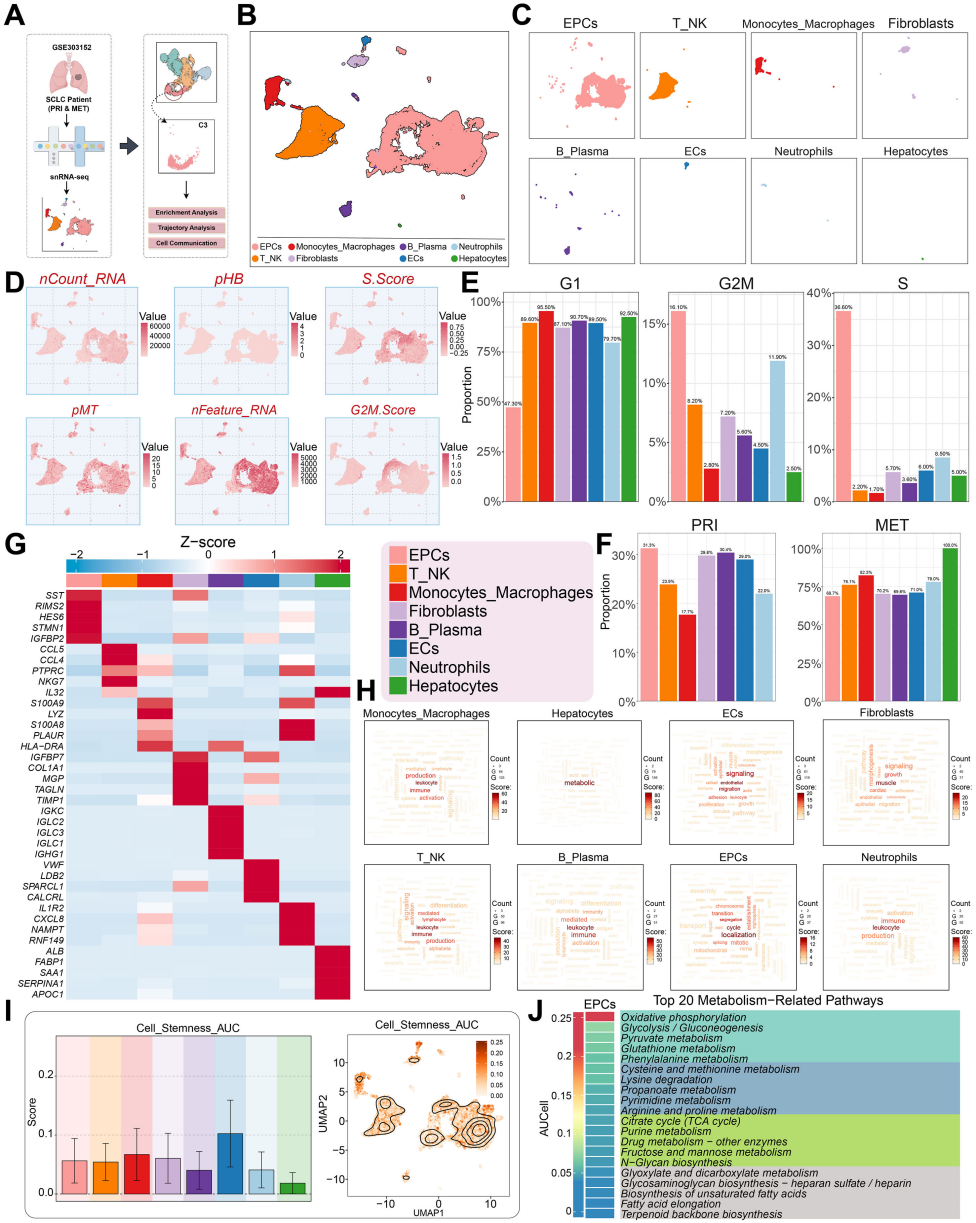
Single-nucleus transcriptomic landscape and heterogeneity analysis of SCLC. **(A)** Flowchart showed the process of further sorting cells to define subpopulations using single-nucleus sequencing. **(B)** UMAP plot showed the distribution of different cell types. **(C)** UMAP plots showed the distribution of different cells separately. **(D)** UMAP plots showed the distribution of cell nCount_RNA, pHB, S.Score, pMT, nFeature_RNA, G2M.Score. **(E)** The bar charts showed the proportion of major cell types in the G1, G2M, and S phases of the cell cycle. **(F)** The bar charts showed the proportion of major cell types in the primary and metastatic groups. **(G)** The heatmap showed the expression levels of the top 5 differentially expressed genes in each cell type. The colors represent the results of data normalization. **(H)** The word cloud diagrams showed the correlation between the major cell types and biological processes. **(I)** The bar chart on the left showed the stemness scores of the major cell types, while the UMAP plot on the right displayed the distribution of stemness scores among the major cell types. **(J)** The heatmap showed the top 20 metabolic pathways in EPCs based on AUCell algorithm scores.

**Figure 1** was the summary diagram of the overall work of this study. In this study, we re-analyzed a single-cell transcriptomic profile of SCLC using data from the GEO database (**Figure 2A**). After processing the single-cell nuclear transcriptomic sequencing data and excluding low-quality cells, we obtained high-quality cells. Based on corresponding marker genes, these cells were classified into diverse major cell clusters, including epithelial cells (EPCs), T/NK cells, monocytes/macrophages, fibroblasts, B/plasma cells, endothelial cells (ECs), neutrophils, and hepatocytes. Among these, EPCs exhibited a broader distribution compared to other cell types (**Figures 2B, C**). By comparing the expression distributions of nCount_RNA, pHB, S.Score, pMT, nFeature_RNA, and G2M.Score in the UMAP plots, we further characterized the heterogeneity of the cells (**Figure 2D**). We then compared the proportion of these cell types across the cell cycle. We observed that EPCs accounted for a lower proportion in the G1 phase compared to other cells, while their proportions in the G2M and S phases were significantly higher than those of other cells, suggesting more active proliferation (**Figure 2E**). Furthermore, by comparing the proportions of these 8 cell types in primary and metastatic samples, we found that in the primary samples, the relative proportion of EPCs was higher than that of other cell types, whereas in the metastasis group, the relative proportion of EPCs was lower than that of other cell types, and hepatocytes were present only in metastatic samples (**Figure 2F**). The heatmap displayed the top 5 genes with the most significant expression differences across these cell types, further confirming the heterogeneity of these cell types (**Figure 2G**). Subsequently, we used word cloud diagrams to illustrate the correlations between the 8 cell types and various biological processes and cellular components. EPCs showed strong correlations primarily with terms such as “localization”, “cycle”, “segregation” and “mitotic”, suggesting that EPCs are closely associated with biological processes such as the cell cycle, DNA replication, and RNA transcription; ECs showed strong correlations with “signaling”, “migration” and “adhesion”, suggesting they may be involved in biological processes such as angiogenesis, migration, adhesion, immune regulation, and signal sensing (**Figure 2H**). Stemness analysis revealed that, among non-immune cells, ECs and EPCs exhibited high stemness (**Figure 2I**). Metabolic pathway analysis indicated that EPCs were primarily associated with metabolic pathways such as oxidative phosphorylation, nucleotide metabolism, and fatty acid synthesis, suggesting that EPCs possess strong proliferative potential (**Figure 2J**). These results collectively demonstrated that EPCs possess relatively high proliferative capacity and high cellular stemness, which may be related to aggressive tumor biology in SCLC.

### 3.2 Visualization of tumor cell subpopulations in SCLC

**Figure 3:**
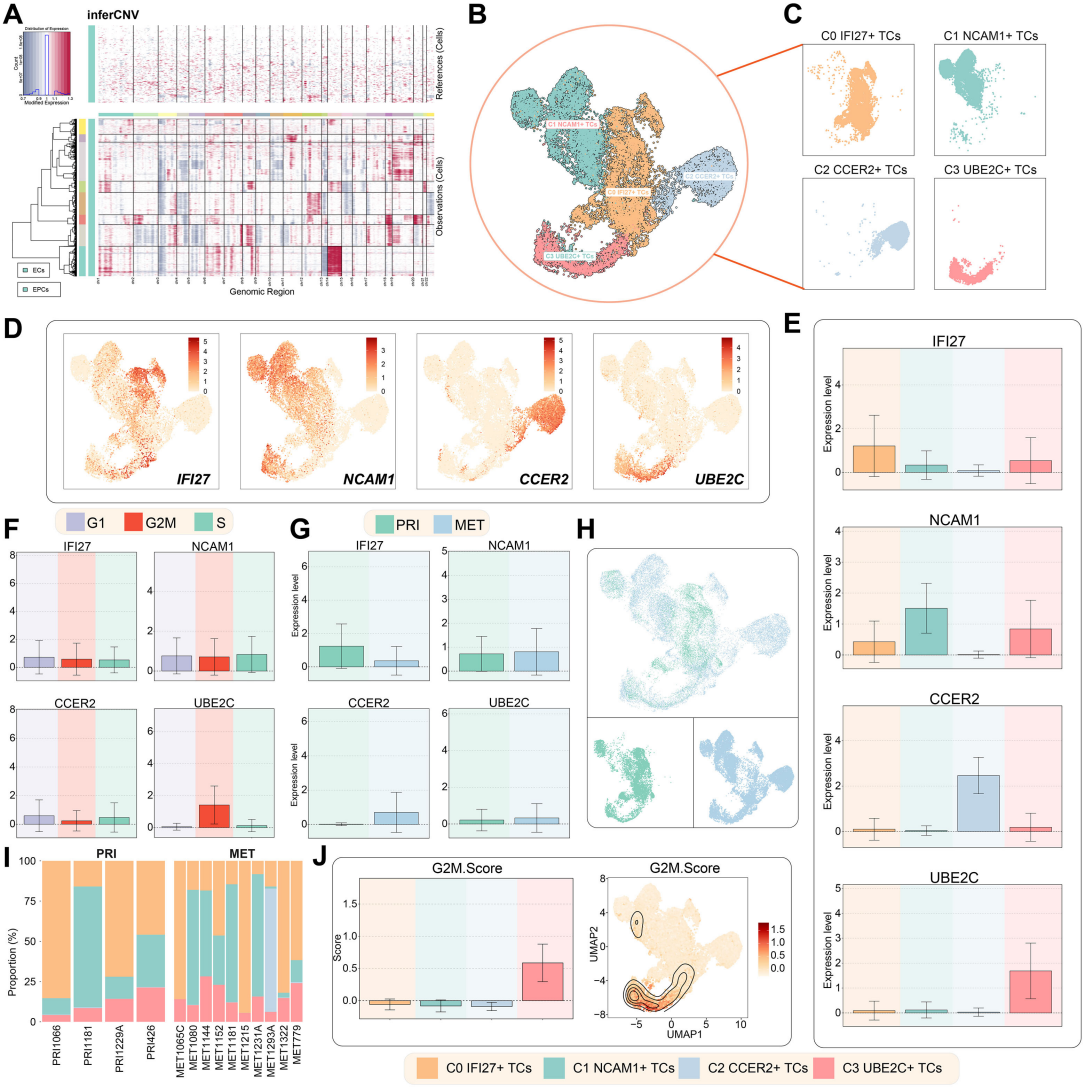
Heterogeneity analysis of tumor cells. **(A)** The inferCNV analysis showed that compared to putative non-malignant ECs reference cells, EPCs exhibited significant expression upregulation (red) or downregulation (blue) in multiple genomic regions, suggesting they may carry CNVs. **(B–C)** UMAP plots showed 4 different tumor subpopulations. **(D)** The UMAP plots showed the expression distribution of 4 tumor cell subpopulations named genes. The color scale represents normalized expression, from white to red: low to high expression. **(E–G)** The bar charts showed the expression distribution of 4 named genes in 4 tumor cell subpopulations, different cell cycles and primary and metastatic groups. **(H)** The UMAP plots showed the expression of tumor cells in the primary and metastatic groups. **(I)** The stacked bar charts showed the proportion of the 4 tumor cell subpopulations in all tumor cells **(J)** The bar chart on the left showed the G2M scores of the 4 tumor cell subpopulations, while the UMAP plot on the right illustrated the value of G2M scores among the tumor cells.

Furthermore, we performed copy number variation analysis on EPCs. The results revealed significant chromosomal copy number differences in multiple genomic regions compared to normal ECs. Based on these differences, we identified malignant tumor cells (**Figure 3A**). Using different named genes, we then classified these malignant tumor cells into 4 subpopulations (**Figures 3B, C**). The distribution of these 4 named genes varied significantly across different tumor cell subpopulations, with *CCER2* expressed almost exclusively in the C2 subpopulation and *UBE2C* expressed almost exclusively in the C3 subpopulation (**Figures 3D, E**). The expression levels of these 4 named genes also varied across different cell cycle phases. From the G1 phase to the S phase, *IFI27* expression gradually decreased, while *NCAM1* and *CCER2* expression first decreased and then increased, and *UBE2C* expression first increased and then decreased suggesting that the C3 subpopulation may possess greater proliferative capacity (**Figure 3F**). Similarly, the expression of these named genes also differed between the primary and metastatic sample groups. *IFI27* expression was higher in the primary sample group, whereas *NCAM1*, *CCER2*, and *UBE2C* expression were higher in the metastatic sample group, which suggested that these subpopulations were enriched in metastatic samples and may warrant further functional investigation (**Figure 3G**). We further compared the expression distribution of tumor cells between the primary and metastatic groups, (**Figure 3H**). We then examined the proportion of the 4 tumor cell subpopulations within the total cell population across different primary and metastatic samples. The proportions of different tumor cell types varied significantly between samples; notably, the C2 subpopulation was detected only in the MET1293A sample. Overall, the C3 subpopulation accounted for a relatively low proportion of all tumor cells, but it was present in both metastatic and primary samples (**Figure 3I**). We also found that the C3 subpopulation exhibited higher G2M scores, suggesting that this subpopulation of tumor cells possesses stronger proliferative capacity (**Figure 3J**). These findings were consistent with the highly proliferative and highly invasive characteristics of SCLC.

### 3.3 Distinct functional and cell-type characteristics of 4 TC subpopulations in SCLC

**Figure 4:**
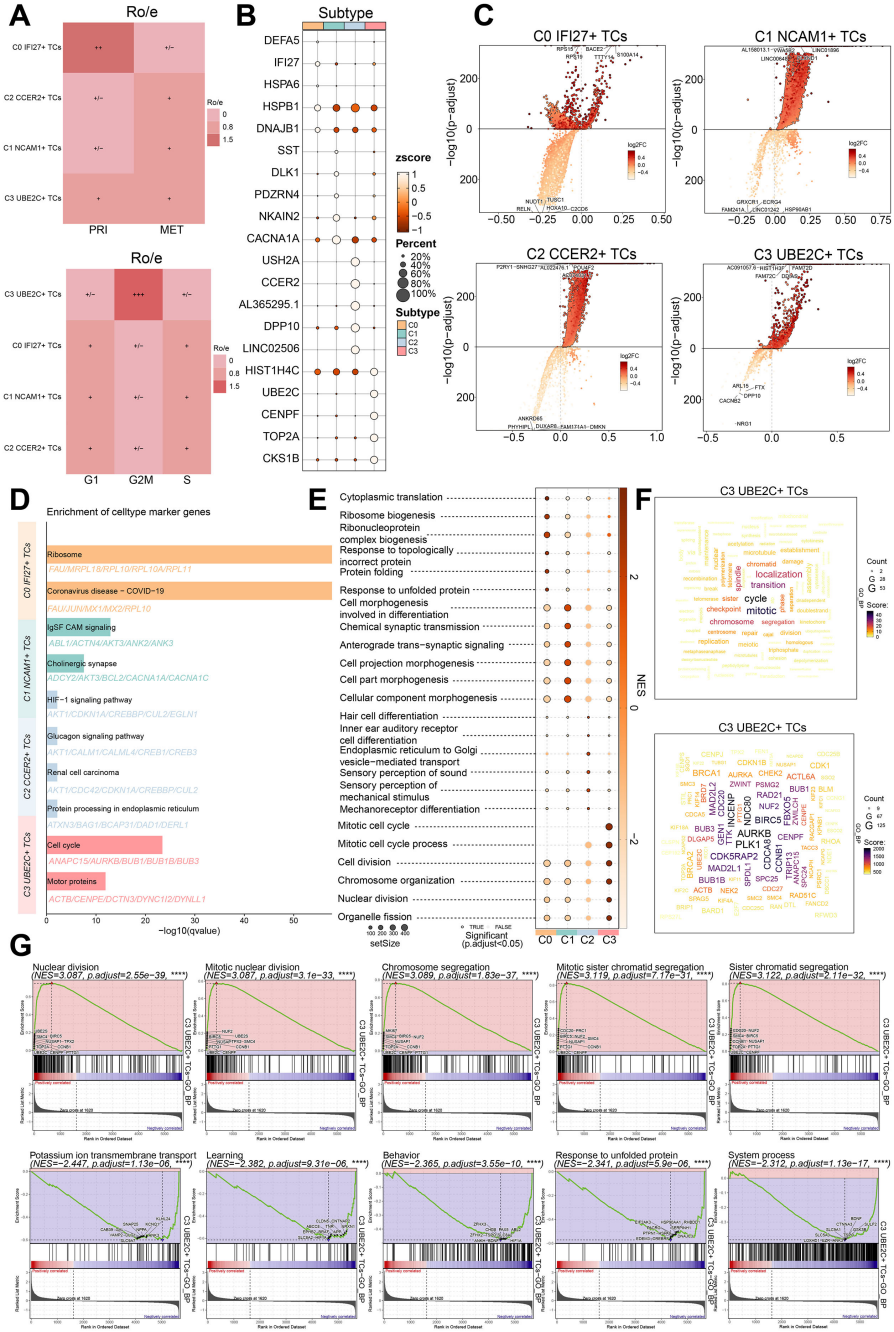
Analysis of distribution preferences, marker gene expression, and functional enrichment characteristics of tumor cell subpopulations. **(A)** The Ro/e preference analysis heatmap showed the distribution preferences of different cell subpopulations in PRI and MET (top panel), as well as their distribution preferences in G1, G2M, and S phases (bottom panel). *+++, R_o/e_ > 1; ++, 0.8 < R_o/e_ ≤ 1; +, 0.2 ≤ R_o/e_ ≤ 0.8; +/−, 0 < R_o/e_ < 0.2; in which R_o/e_ denoted the ratio of observed to expected cell number.* **(B)** The bubble chart showed the expression levels of the top 5 DEGs in the 4 cell subpopulations. **(C)** The volcano plots showed the differentially upregulated and downregulated genes in each cell subpopulation. **(D)** The KEGG enrichment analysis chart showed the enriched pathways of cell subpopulations. **(E)** The bubble chart showed the enriched pathways for each cell subpopulation. **(F)** The word cloud diagrams showed the biological processes and genes enriched in C3 *UBE2C*+ TCs. **(G)** GSEA enrichment analysis showed the upregulated and downregulated pathways in the C3 subpopulation. ****P < 0.0001.

To further understand the distribution of the 4 subpopulations across different tissue types and cell cycle phases, we performed Ro/e analysis. The results showed that C0 *IFI27*+ TCs accounted for cell abundance in the primary tumor group, C1 and C2 subpopulations had a greater preference for the metastatic group. In the cell cycle preference analysis, C3 **UBE2C*+* TCs had a greater preference in the G2M phase, suggesting active proliferation (**Figure 4A**). We then analyzed the top 5 DEGs for each of the 4 subpopulations: for the C0 subpopulation, these were *DEFA5*, *IFI27*, *HSPA6*, *HSPB1*, and *DNAJB1*; the C1 subpopulation included *SST*, *DLK1*, *PDZRN4*, *NKAIN2*, and *CACNA1A*; the C2 subpopulation included *USH2A*, *CCER2*, *AL365295.1*, *DPP10*, and *LINC02506*; the C3 subpopulation included *HIST1H4C*, *UBE2C*, *CENPF*, *TOP2A*, and *CKS1B* (**Figure 4B**), confirming the heterogeneity of the 4 tumor cell subpopulations. Volcano plots displayed the upregulated and downregulated genes in each cell subpopulation (**Figure 4C**). KEGG enrichment analysis reflected the functional heterogeneity among cell subpopulations (**Figure 4D**). The C3 subpopulation was primarily involved in cell cycle-related pathways. GSEA analysis indicated that the C3 subpopulation highly expresses cell division-related pathways in SCLC, suggesting that this subpopulation was proliferatively active and promotes tumor growth (**Figure 4E**). Combined with the preceding analyses, this demonstrated that C3 **UBE2C*+* TCs played a crucial role in the progression of SCLC. The word cloud diagrams further illustrated that the C3 subpopulation was enriched in biological process and genes such as cycle, mitotic, *AURKB*, and *PLK1*, further corroborating their tumor-promoting characteristics (**Figure 4F**). Additionally, GSEA enrichment analysis suggested that gene expression promoting rapid cell proliferation was significantly upregulated in C3 *UBE2C*+ TCs (**Figure 4G**). In summary, C3 *UBE2C*+ TCs accounted for a higher proportion in the metastatic tumor and were predominantly in the G2M phase. They highly expressed mitosis-related genes and were enriched in mitosis-related pathways. These findings suggested that they represent a key subpopulation that is actively proliferating and promotes the progression of SCLC.

### 3.4 Metabolic and stemness of cell characteristics of 4 subpopulations

**Figure 5:**
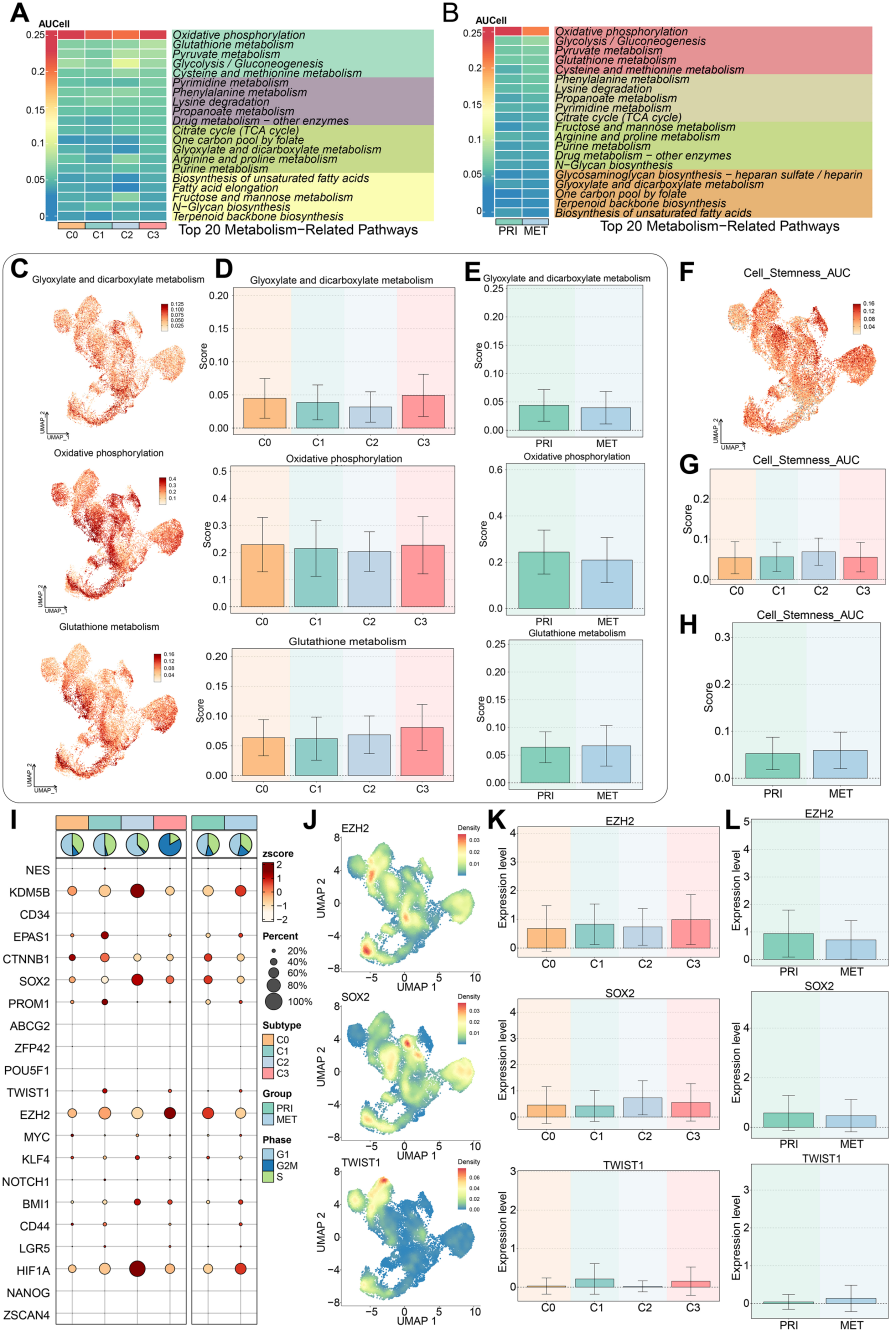
Gene expression and functional enrichment of tumor cell subpopulations. **(A)** Comparison of AUCell scores for the top 20 metabolism-related pathways among the 4 tumor cell subpopulations. **(B)** Comparison of AUCell scores for the top 20 metabolic pathways in the primary and metastatic groups. **(C)** The UMAP plots showed the distribution of metabolic activity for the three pathways: Glyoxylate and dicarboxylate metabolism, Oxidative phosphorylation, and Glutathione metabolism. **(D)** The bar charts showed the comparison of metabolic activity of the Glyoxylate and dicarboxylate metabolism, Oxidative phosphorylation, and Glutathione metabolism pathways among the 4 subpopulations C0-C3. **(E)** The bar charts showed a comparison of the metabolic activities of three pathways, Glyoxylate and dicarboxylate metabolism, Oxidative phosphorylation, and Glutathione metabolism-between primary and metastatic sample groups. **(F)** The UMAP plot showed the distribution of stemness scores in tumor cells. **(G–H)** The bar charts displayed the stemness scores of the 4 subpopulations and different groups. **(I)** The bubble chart showed the expression levels of stemness markers in different subpopulations and different groups. **(J)** The UMAP plots showed the density of the three genes EZH2, SOX2, and TWIST1 in the cells. **(K)** The bar charts respectively showed the expression levels of the three genes EZH2, SOX2, and TWIST1 in the 4 subpopulations. **(L)** The bar charts respectively showed the expression levels of the three genes EZH2, SOX2, and TWIST1 in primary and metastatic group samples.

To further investigate the characteristics of tumor cells, we performed metabolic pathway analyses across different subpopulations and sample groups. The results showed that the C3 **UBE2C*+* TCs subpopulation exhibited higher activity in the oxidative phosphorylation, glycolysis/gluconeogenesis, and glutathione metabolism pathways (**Figure 5A**). Additionally, there were significant differences in metabolic pathway activity between the primary and metastatic groups (**Figure 5B**). Furthermore, we investigated the differences in the distribution and expression of the three pathways across different subpopulations and sample groups including glyoxylate and dicarboxylate metabolism, oxidative phosphorylation, and glutathione metabolism. The results showed that the C3 *UBE2C*+ TCs subpopulation exhibited a synergistic enhancement of oxidative phosphorylation and glutathione metabolism, suggesting that this subpopulation may rely on mitochondrial oxidative metabolism to maintain energy supply while countering proliferation-related oxidative stress through strong antioxidant defenses. Notably, oxidative phosphorylation activity was higher in the primary tumor group than in the metastatic group, while glutathione metabolism levels were similar between the two groups, suggesting that the metastatic process may be accompanied by metabolic reprogramming (**Figures 5C–E**). We subsequently analyzed and compared cellular stemness across different subpopulations and sample groups. We found that the C3 subpopulation exhibited lower stemness than the C2 subpopulation, with no significant differences observed between the C0 and C1 subpopulations (**Figures 5F, G**), whereas the metastatic group had higher stemness scores than the primary sample group (**Figure 5H**). The expression profile of stemness genes further revealed that tumor cells highly express *EZH2*, *SOX2*, and *TWIST1* (**Figures 5I, J**), forming a unique stemness regulatory network; among these, *EZH2* expression was highest in the C3 subpopulation, *SOX2* expression was highest in the C2 subpopulation; *TWIST1* expression was highest in the C1 subpopulation and was almost undetectable in C0 and C2 (**Figure 5K**). Compared to the primary tumor group, *EZH2* and *SOX2* were expressed at lower levels in the metastatic group, whereas *TWIST1* was expressed at higher levels in the metastatic group (**Figure 5L**). In summary, the metabolic and stemness of cell characteristics of tumor cell subpopulations exhibited heterogeneity and dynamic plasticity.

### 3.5 Pseudotime analysis revealed developmental and differentiation characteristics of TC subpopulations

**Figure 6:**
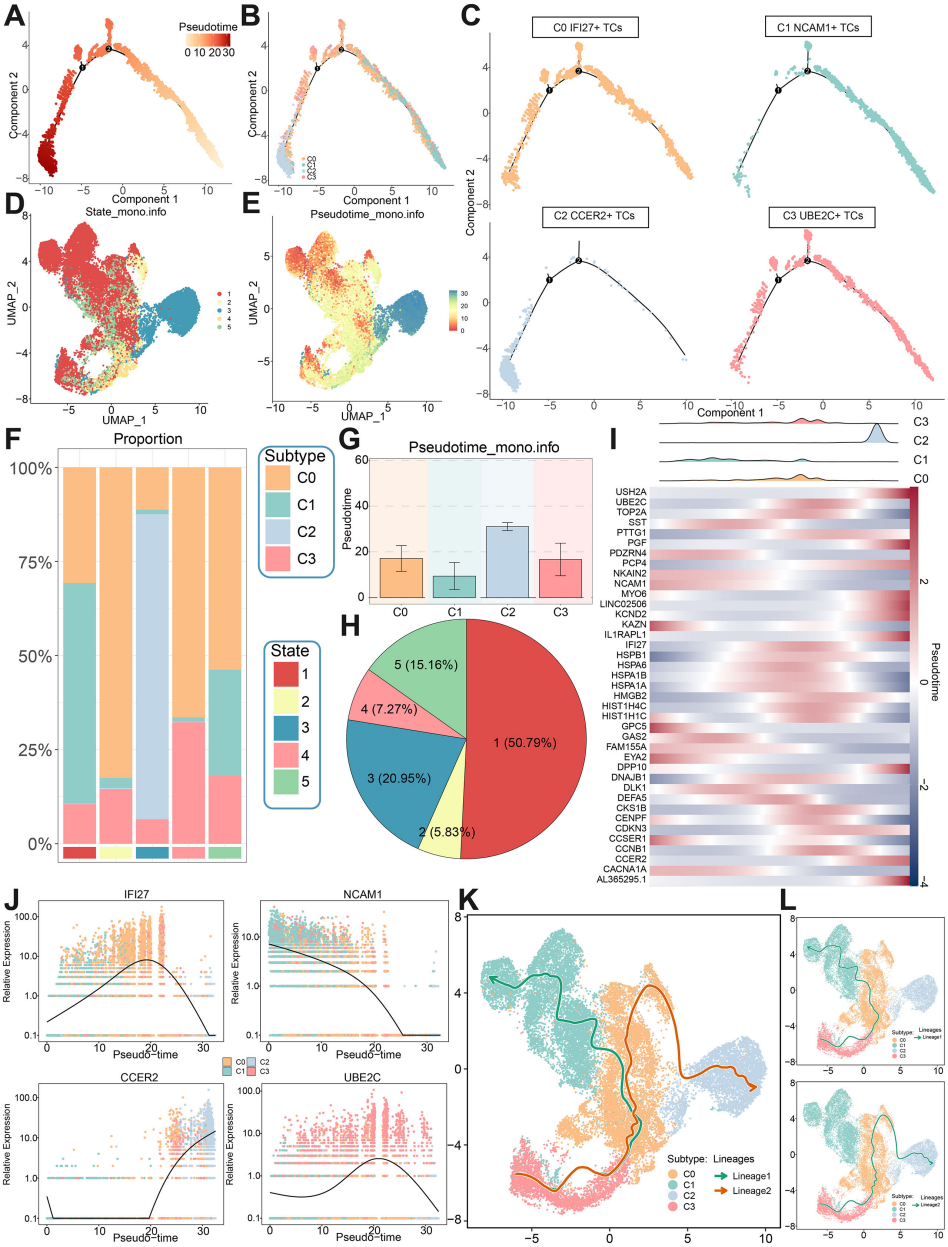
Pseudotime trajectory analysis of tumor cell subpopulations. **(A)** Trajectory analysis presented the development process of the pseudotime trajectory. **(B)** Trajectory analysis showed the distribution of all tumor cell subpopulations along the pseudotime trajectory. **(C)** Trajectory analysis showed various tumor cell subpopulations under different pseudotime states. **(D)** The UMAP plot showed the distribution of cells in different states. **(E)** The UMAP plot showed the dynamic evolution process of cells along the pseudotime trajectory. **(F)** The stacked bar chart showed the proportion of different cell subpopulations in each differentiation state. **(G)** The bar plot showed the pseudotime distribution characteristics of different cell subpopulations. **(H)** The pie chart showed the distribution of each cell state within the overall cell population. **(I)** The heatmap showed the expression patterns of key genes along the pseudotime trajectory, with the upper curve displaying the distribution density of each cell subpopulation along the pseudotime axis. **(J)** The scatter plots showed the dynamic expression changes of the named genes *IFI27*, *NCAM1*, *CCER2*, and *UBE2C*. **(K)** The UMAP plot showed the differentiation trajectories of the 4 subpopulations. The solid line represents the differentiation trajectory, and the arrows indicate the direction of differentiation. **(L)** The UMAP plots showed two distinct cell differentiation trajectories.

To elucidate the lineage evolution and subpopulation development differences in SCLC tumor cells, we performed pseudotime trajectory analysis using monocle and slingshot. Pseudotime ordering analysis based on monocle identified the relative pseudotime positions of each subpopulation, with the determined pseudotime trajectories extending from the right (early) to the left (late) (**Figure 6A**). Pseudotime mapping of tumor subpopulations revealed that C1 *NCAM1*+ TCs were predominantly distributed in the early pseudotime, C0 *IFI27*+ TCs and C3 **UBE2C*+* TCs were both distributed throughout, while C2 *CCER2*+ TCs were concentrated in the late stage (**Figures 6B, C**). We classified tumor cells into 5 states based on trajectory branching points, where state 1 represents the initial pseudotime phase, state 3 represents the late pseudotime phase, and the other states covered the early, intermediate, and late pseudotime phases (**Figures 6D, E**). UMAP dimension reduction results further validated the dominant distribution of state 1, the independent clustering of state 3, and the transitional patterns of other subpopulations. State 1 was dominated by C0 *IFI27*+ TCs and C1 *NCAM1*+ TCs; state 2 exhibited enrichment of C0 *IFI27*+ TCs; state 3 was primarily composed of C2 *CCER2*+ TCs; state 4 was dominated by C0 *IFI27*+ TCs and C3 **UBE2C*+* TCs; state 5 exhibited enrichment of C0 *IFI27*+ TCs, followed sequentially by C1 *NCAM1*+ TCs and C3 **UBE2C*+* TCs (**Figure 6F**). Bar plot results indicated significant differences in the pseudotime distributions of the C0, C1, C2, and C3 subpopulations: the pseudotime values of the C2 subpopulation were significantly higher than those of the other subpopulations, suggesting it was at the terminal stage of the differentiation pathway (**Figure 6G**). Statistical analysis of the proportion of tumor cell subpopulations by cell state revealed that state 1 was the dominant subpopulation (50.79%), followed by state 3 (20.95%) and state 5 (15.16%), while state 4 (7.27%) and state 2 (5.83%) accounted for a lower proportion, suggesting significant heterogeneity in the distribution of tumor cells in different states within the cell population (**Figure 6H**). To elucidate the molecular dynamics during tumor cell differentiation, we visualized gene expression patterns from the pseudotime series analysis using heatmaps. The results showed that gene expression exhibited significant temporal dynamics over the pseudotime course and could be divided into multiple expression modules: during the early differentiation stage; during the mid-differentiation stage (corresponding to the C0/C3 subpopulations), genes associated with cellular functional specialization (*UBE2C*, *PTTG1*) were gradually upregulated; and during the late differentiation stage, genes such as *MYO6*, *LINC02506*, and *KCND* are upregulated, showing distinct stage-specific enrichment; The gene expression patterns of different subpopulations align closely with the pseudotime progression, suggesting that tumor cells undergo transcriptional remodeling from a basal state to a functionally specialized state during differentiation (**Figure 6I**). Trends in named gene expression indicated that *CCER2* expression increases toward the end of the trajectory; *NCAM1* dominates at the beginning of the trajectory; while *IFI27* and *UBE2C* reach their peaks in the middle segment (**Figure 6J**). Subsequently, we used the slingshot analysis to reconstruct two lineage trajectories, named Lineage1 and Lineage2: Lineage1: C3 **UBE2C*+* TCs → C0 *IFI27*+ TCs → C1 *NCAM1*+ TCs; Lineage2: C3 **UBE2C*+* TCs → C0 *IFI27*+ TCs → C2 *CCER2*+ TCs (“→” denoted a time series) (**Figures 6K, L**). In summary, the C3 subpopulation occupied a crucial position in the cellular differentiation lineage and participates in lineage differentiation. Therefore, these trajectory analyses placed C3 *UBE2C*+ TCs in an important state, suggesting that this subpopulation may be involved in tumor cell state transitions. However, tumor cells rarely act in isolation within the tumor immune microenvironment, where abnormal intercellular signaling frequently occurs. For this reason, we conducted further analysis.

### 3.6 Cell-cell communication analysis identified GRN and THBS signaling associated with *UBE2C*+ TCs

**Figure 7:**
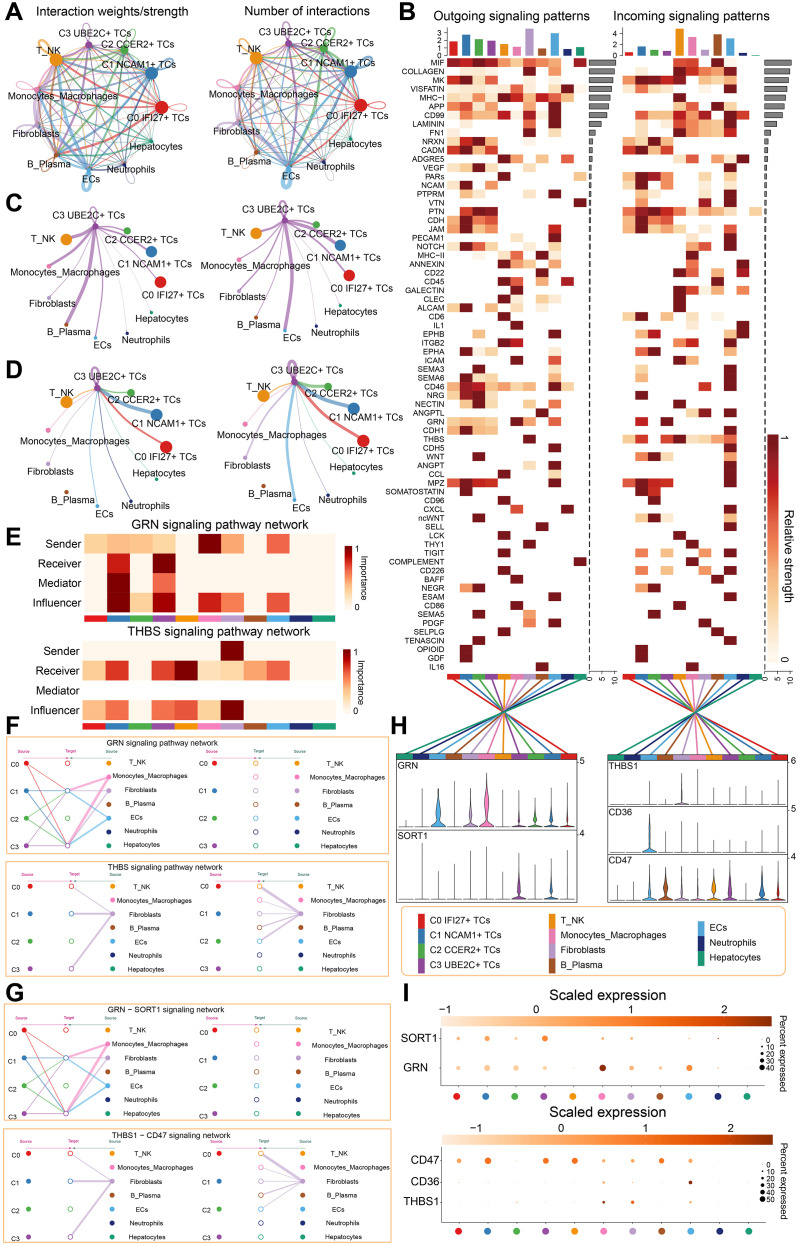
Intercellular communication and interactions. **(A)** The circle charts showed the intensity (left) and quantity (right) of communication between cell types. **(B)** Heatmaps showed the signaling pathway activity of different cell types, with outgoing signals on the left and incoming signals on the right. **(C)** The circle diagrams reflected the intensity (left) and quantity (right) of the C3 subpopulation as a signal sender with other cell subpopulations. **(D)** The circle diagrams reflected the intensity (left) and quantity (right) of the C3 subpopulation as signal receivers with other cell subpopulations. **(E)** Heatmaps showed the centrality scores of the GRN signaling pathway (top) and the THBS signaling pathway (bottom). **(F)** The hierarchical diagrams illustrated the interaction relationships between different cell types in the GRN (top) and THBS (bottom) signaling pathways. **(G)** The hierarchical communication diagrams showed the intercellular signaling mediated by the GRN-SORT1 ligand-receptor pair (top) and the THBS1-CD47 ligand-receptor pair (bottom). **(H)** The violin plots showed the distribution of expression levels of key ligands (GRN, THBS1) and receptors (SORT1, CD36, CD47) across different cell types. The width of the violin plots indicated the density of expression levels, with different colors corresponding to different cell types. **(I)** The bubble charts showed the gene expression levels in the GRN-SORT1 ligand-receptor (top) and the THBS1-CD47/CD36 ligand-receptor (bottom).

To systematically analyze the characteristics of intercellular communication mediated by different tumor cell subpopulations in the microenvironment of SCLC, we constructed a multi-cell ligand-receptor interaction network based on CellChat analysis. We used circle plots to illustrate the intensity and number between cell subpopulations (**Figure 7A**). We then compared the intensity of different signaling pathways in the signaling and reception patterns among cell subpopulations and found that signaling pathways such as MIF and LAMININ exhibited higher expression levels (**Figure 7B**). To further investigate the key role of C3 *UBE2C*+ TCs in tumor progression and their potential functional synergies, we used circle plots to visualize the intensity and number of interactions between the C3 subpopulation and other cell types when acting as both a signal sender and receiver: when the C3 subpopulation acted as a signal sender, the C3 subpopulation exhibited active cellular communication with the C1 subpopulation, B/Plasma, and ECs; when acting as a signal receiver, the C3 subpopulation exhibited active cellular communication with the C0, C1 subpopulations, and fibroblasts (**Figures 7C, D**). In the GRN signaling pathway, macrophages/monocytes act as the signal sender through high expression of GRN, whereas C3 *UBE2C*+ tumor cells serve as the main recipients via expression of the receptor SORT1; In the centrality analysis of the THBS signaling network, C3 *UBE2C*+ TCs acted as both recipients and regulators, while fibroblasts function as signal transmitters and regulators, which may be associated with promoting tumor progression (**Figure 7E**). C3 *UBE2C*+ TCs might interact with macrophages; simultaneously, C3 *UBE2C*+ TCs might interact with fibroblasts (**Figure 7F**), thereby establishing significant communication strength between these two cell populations. Hierarchical network analysis inferred potential GRN-SORT1 communication between C3 *UBE2C*+ TCs and monocytes/macrophages, and potential THBS1-CD47 communication between C3 *UBE2C*+ TCs and fibroblasts (**Figure 7G**). Violin plots demonstrated that the ligand GRN was highly expressed in macrophages, while the ligand THBS1 was highly expressed in fibroblasts; receptor SORT1 was highly expressed in C3 *UBE2C*+ TCs, and receptor CD47 was highly expressed in C3 *UBE2C*+ TCs (**Figure 7H**). THBS1-CD47 was identified as the primary ligand-receptor interaction between stromal fibroblasts and C3 *UBE2C*+ tumor cells, while CD36 also exhibited detectable but comparatively weaker interaction signals, suggesting a potential auxiliary receptor role in this signaling network. The bubble plot indicated that C3 *UBE2C*+ TCs may interact with macrophages via ligand GRN and receptor SORT1; C3 **UBE2C*+* TCs may interact with fibroblasts via the ligand THBS1 and the receptor CD47(**Figure 7I**). This study provided deep insights into the complex interaction mechanisms between tumor cell subpopulations and macrophages and fibroblasts in SCLC. This relationship was likely closely associated with driving the progression of SCLC.

### 3.7 Identification and analysis of TF regulatory modules

**Figure 8:**
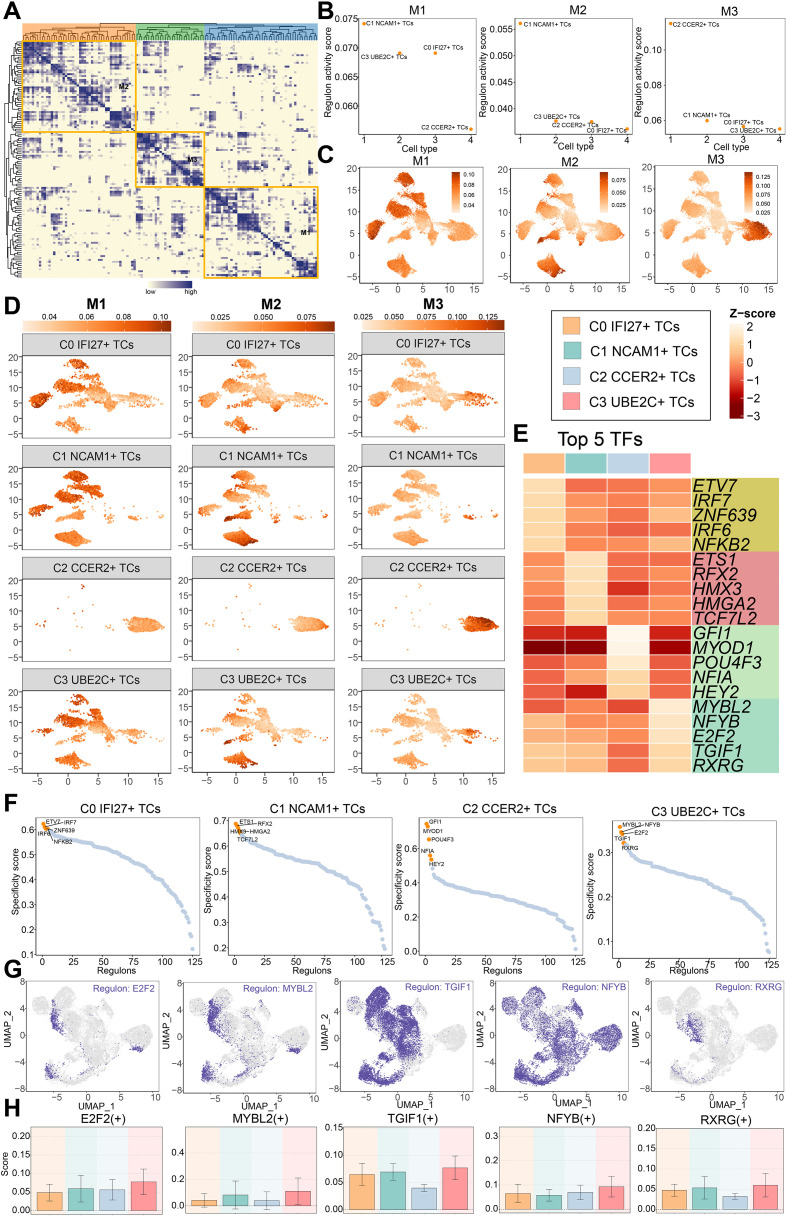
Identification of gene regulatory networks in C3 *UBE2C*+ TCs. **(A)** The heatmap showed the identification of three regulatory modules in SCLC cell subpopulations based on SCENIC regulatory rule modules and AUCell similarity scores. **(B)** The scatter plots showed the expression of C0 to C3 subpopulations in different modules. **(C)** The UMAP plots showed the expression of the three modules in the cell subpopulations. **(D)** The facet plots respectively showed the value of M1, M2, and M3 modules in different subpopulations. **(E)** The heatmap respectively showed the top 5 TFs in the 4 subpopulations. **(F)** The scatter plots showed the expression ranking of the Top 5 TFs in the tumor cell subpopulations. **(G-H)** The UMAP plots and bar charts respectively showed the distribution characteristics and transcriptional activity of the top 5 TFs in the 4 subpopulations.

To investigate the mechanisms underlying the progression of SCLC and identify potential therapeutic targets, we classified cells into three TF modules based on a connection specificity index (CSI) matrix (**Figure 8A**). Analysis of the expression patterns of different cell subpopulations across the three modules revealed that the C3 subpopulation exhibited higher expression levels in the M1 module (**Figure 8B**). Furthermore, we visualized the overall expression distribution of M1, M2, and M3 across cell populations using UMAP plots (**Figure 8C**) and presented the expression status of subpopulations C0 to C3 in the three modules via facet plots (**Figure 8D**). Heatmaps displayed the top 5 TFs in each subpopulation, revealing that MYBL2, NFYB, E2F2, TGIF1, and RXRG were the 5 most highly expressed TFs in the C3 subpopulation (**Figure 8E**). Furthermore, scatter plots of TF expression levels across each cell subpopulation were consistent with these findings (**Figure 8F**). Subsequently, we focused on the C3 subpopulation, visualizing the distribution of these 5 TFs using UMAP plots and verifying the transcriptional activity of MYBL2, NFYB, E2F2, TGIF1, and RXRG via bar charts (**Figures 8G, H**). Notably, the expression of E2F2 and TGIF1 in the C3 subpopulation was significantly higher than in other subpopulations. In summary, this study classified cells into three TF modules using a CSI matrix and systematically revealed the expression profiles of each cell subpopulation across different modules. The results indicated that the C3 subpopulation is significantly highly expressed in the M1 module and possesses a unique TF regulatory network. These findings provided important clues for a deeper understanding of the progression mechanisms of SCLC and the identification of new therapeutic targets.

### 3.8 *UBE2C* knockdown suppressed proliferative and migratory phenotypes in SCLC cells

Single-nucleus transcriptomic analysis identified a tumor cell subpopulation characterized by elevated proliferative and cell cycle–associated transcriptional programs in SCLC. Among the differentially expressed genes enriched in this malignant tumor cell population, *UBE2C* was selected for functional validation because of its established role in mitotic progression and tumor aggressiveness.

**Figure 9:**
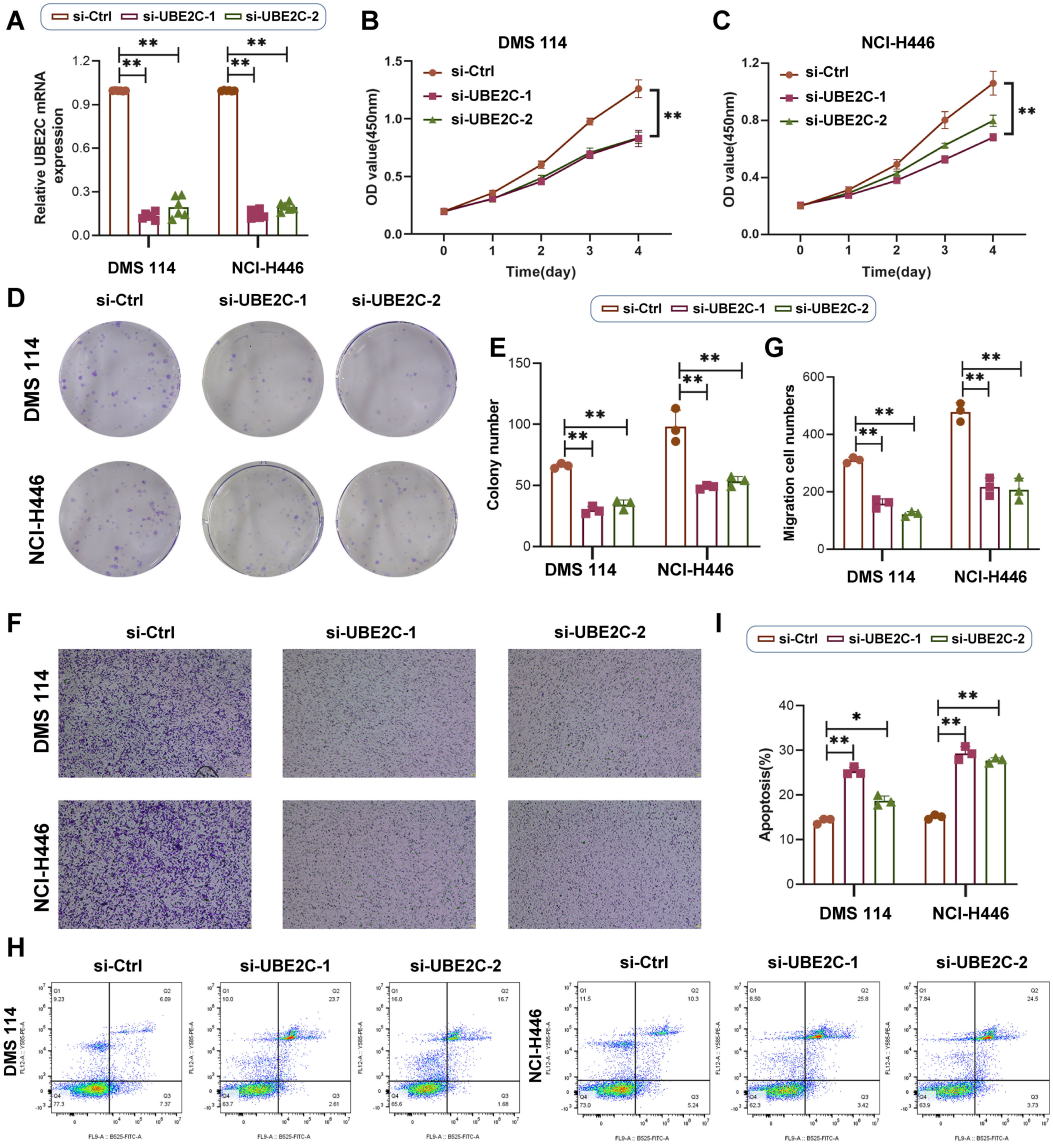
*In vitro* cell experiments. **(A)** qRT-PCR analysis validated *UBE2C* knockdown efficiency in DMS114 and NCI-H446 cells transfected with si-*UBE2C*-1 or si-*UBE2C*-2 compared with si-Ctrl. **(B-C)** CCK-8 assays showed cell viability changes following *UBE2C* silencing in DMS114 **(B)** and NCI-H446 **(C)** cells, measured by OD450 values over time. **(D)** Representative images of colony formation assays in DMS114 and NCI-H446 cells after *UBE2C* knockdown. **(E)** Quantification of colony numbers showing reduced clonogenic ability following *UBE2C* depletion. **(F)** Representative images of transwell migration assays demonstrating decreased migratory capacity after *UBE2C* silencing. **(G)** Quantification of migrated cell numbers in DMS114 and NCI-H446 cells. **(H)** Representative Annexin V/PI flow cytometry plots showing apoptosis in indicated groups. **(I)** Quantitative analysis of apoptotic cell percentages. Data were presented as mean ± SD from three independent experiments. Statistical significance was determined using one-way ANOVA followed by multiple-comparison testing. *P < 0.05, **P < 0.01.

To investigate the biological function of *UBE2C* in SCLC, two human SCLC cell lines, DMS114 and NCI-H446, were transfected with two independent siRNAs targeting *UBE2C* (si-*UBE2C*-1 and si-*UBE2C*-2) or a non-targeting control siRNA (si-Ctrl). qRT-PCR analysis confirmed efficient *UBE2C* mRNA depletion in both cell lines following siRNA transfection (**Figure 9A**).

We next evaluated the effect of *UBE2C* depletion on short-term proliferative capacity using CCK-8 assays. Silencing *UBE2C* significantly suppressed the time-dependent increase in OD_450_ values in both DMS114 and NCI-H446 cells compared with si-Ctrl cells (**Figures 9B, C**), indicating impaired proliferative activity following *UBE2C* knockdown.

To further assess long-term proliferative potential, colony formation assays were performed. *UBE2C* depletion markedly reduced colony numbers in both SCLC cell lines (**Figures 9D, E**), suggesting that *UBE2C* contributes to sustained clonogenic growth capacity.

Because aggressive tumor progression was frequently associated with enhanced migratory ability, we next performed transwell migration assays to determine whether *UBE2C* regulates SCLC cell motility. Compared with control cells, silencing *UBE2C* significantly decreased the migration capacity of both DMS114 and NCI-H446 cells (**Figures 9F, G**), indicating that *UBE2C* may promote migratory phenotypes in SCLC cells.

We further examined whether *UBE2C* affected apoptotic cell death using Annexin V/PI flow cytometry. Knockdown of *UBE2C* significantly increased the proportion of apoptotic cells in both DMS114 and NCI-H446 cells compared with si-Ctrl cells (**Figures 9H, I**), indicating that *UBE2C* may contribute to SCLC cell survival.

Collectively, these findings demonstrated that si-*UBE2C* suppressed proliferation, clonogenic growth, migration, and survival of SCLC cells, supporting its role as a potential oncogenic driver in SCLC.

## 4 Discussion

In this study, we applied an integrative snRNA-seq-based systems biology framework to dissect malignant cell states and tumor-immune regulatory crosstalk in SCLC. We identified 4 transcriptionally distinct malignant tumor cell subpopulations and highlighted C3 **UBE2C*+* TCs as a proliferative state characterized by G2/M enrichment, mitotic gene expression, metabolic activation, and a transitional position along pseudotime trajectories. Importantly, this subpopulation was not only defined by tumor-intrinsic proliferative programs but was also connected to macrophage- and fibroblast-associated communication networks, including GRN-SORT1 and THBS1-CD47/CD36 signaling. Functional knockdown experiments further confirmed that si-*UBE2C* suppressed SCLC cell proliferation, clonogenicity, migration, and survival. Together, these findings suggest that **UBE2C*+* TCs may represent a regulatory node linking malignant proliferation to the immune-stromal microenvironment in SCLC.

The metabolic features of C3 **UBE2C*+* TCs also provide insight into their functional state. Enhanced oxidative phosphorylation and glutathione metabolism suggest that this subpopulation may require increased mitochondrial energy production while simultaneously maintaining antioxidant capacity to tolerate proliferation-associated oxidative stress. Such metabolic coupling may represent an adaptive mechanism that supports rapid tumor expansion. Because metabolic programs can influence immune cell function and therapeutic response, future studies integrating metabolomics or spatial metabolic imaging may clarify whether the metabolic state of **UBE2C*+* TCs contributes to immune suppression or treatment resistance.

Trace analysis indicated that the C3 **UBE2C*+* TCs subpopulation is primarily distributed during the early to mid-stages of lineage development. *UBE2C* was not only a molecular marker for this subpopulation but was also likely the core effector molecule responsible for maintaining its proliferative activity and executing its functions. As an E2 ubiquitin ligase, *UBE2C* acts in concert with the anaphase-promoting complex/cyclosome (APC/C) to specifically regulate the degradation of key cell cycle proteins (such as Cyclin B, Securin, and PLK1), thereby driving cells from the G2 phase into the M phase and maintaining the high proliferative potential of the C3 subpopulation (63–65). At the same time, *UBE2C* can inhibit autophagy by activating the Akt-mTOR signaling pathway, thereby enhancing adaptability to changes in the immune microenvironment and ensuring cell survival (66).

A key feature of this study is the integration of malignant cell state analysis with tumor microenvironmental communication inference. CellChat analysis suggested that C3 *UBE2C*+ TCs may receive GRN signals from monocytes/macrophages through SORT1 and may also participate in THBS1-CD47/CD36-mediated communication with fibroblasts. These predicted signaling interactions suggested potential links with tumor-promoting microenvironmental processes, including macrophage- associated regulation, stromal remodeling, and immune evasion. Therefore, the predicted GRN-SORT1 and THBS1-CD47/CD36 axes may provide mechanistic links between proliferative tumor cells, myeloid cells, fibroblasts, and immune escape in SCLC. Although these interactions require experimental validation, they nominate candidate regulatory circuits through which the immune-stromal niche may support a highly proliferative tumor state.

TF module analysis further expanded the regulatory interpretation of the C3 *UBE2C*+ TCs state. The enrichment of MYBL2, NFYB, E2F2, TGIF1, and RXRG suggests that proliferative SCLC cells may be maintained by coordinated transcriptional programs rather than by isolated marker genes. In particular, E2F family members are canonical regulators of cell cycle entry and DNA replication, while MYBL2 is frequently associated with mitotic progression and aggressive tumor behavior. These findings support a model in which **UBE2C*+* TCs are embedded within a broader cell cycle-centered transcriptional regulome. Future perturbation experiments targeting these transcriptional regulators, alone or in combination with *UBE2C* inhibition, may help identify synthetic vulnerabilities in SCLC.

In *UBE2C* knockdown experiments, we evaluated its effects on proliferation, migration, clonogenic growth, and apoptosis. The CCK-8 assay showed that the short-term proliferation capacity of *UBE2C*-knockdown cells was significantly reduced, while the colony formation assay indicated that their long-term clonal growth was also inhibited. In addition, the transwell assays confirmed that the migration capacity of *UBE2C*-knockdown cells was markedly reduced. Finally, flow cytometry revealed a significant increase in the apoptosis rate of *UBE2C*-knockdown cells. Therefore, we have preliminarily demonstrated that *UBE2C* played a critical role in cell proliferation, colony formation, cell migration, and the suppression of apoptosis.

*UBE2C* has also been implicated in other types of lung cancer. Previous studies have reported that *UBE2C* is frequently upregulated in lung adenocarcinoma and lung squamous cell carcinoma, where it is associated with enhanced tumor proliferation, poor prognosis, and increased cell cycle activity (67). Mechanistically, *UBE2C* has been shown to promote malignant progression primarily through dysregulation of the APC/C-mediated cell cycle control and mitotic checkpoint pathways (68). These findings are consistent with its role observed in SCLC in the present study, suggesting that *UBE2C* may represent a broadly conserved regulator of cell cycle-driven tumor aggressiveness across different lung cancer subpopulations.

Single nucleus sequencing technology has overcome the limitations of traditional population-based transcriptomic sequencing, enabling us to analyze the heterogeneity of tumor cells in SCLC at single-cell resolution. By mapping the cellular interaction networks between tumor cells and immune/stromal cells, we have revealed the specific interaction mechanisms between subpopulations and the microenvironment, providing a new perspective for understanding the complex molecular mechanisms of SCLC. The inclusion of *in vitro* experiments further validated the role of *UBE2C* as a key gene.

Importantly, the snRNA-seq dataset (GSE303152) analyzed in this study was originally generated by a recent Nature (2025) study focusing on neuronal activity-dependent mechanisms in SCLC pathogenesis and tumor–neuron interactions (10). In contrast, the present study re-analyzes the same dataset from a tumor cell–centric perspective, with a specific emphasis on malignant cell heterogeneity, lineage-related transcriptional states, and tumor microenvironmental communication networks. While the original work primarily highlighted neurobiological signaling as a key driver of SCLC progression, our analysis further delineates a proliferative *UBE2C*+ tumor cell state and its associated intercellular signaling pathways, providing an additional layer of interpretation of intratumoral cellular hierarchy. Together, these findings extend the functional understanding of SCLC by complementing the original neurobiological framework with a detailed tumor heterogeneity and cell-state–oriented perspective.

Nevertheless, several limitations should be acknowledged. First, this study is based on a single publicly available snRNA-seq dataset (GSE303152), which may limit the generalizability of the findings. Small cell lung cancer (SCLC) is highly heterogeneous both inter- and intra-patient, and significant biological variability exists across different cohorts, clinical stages, and treatment backgrounds. Therefore, conclusions drawn from a single dataset may not fully capture the global transcriptional landscape of SCLC. Second, as this analysis relies on retrospective public data, unavoidable selection bias and technical variability may still exist despite batch correction using Harmony.Third, malignant cell annotation and subpopulation definition were primarily inferred using computational approaches such as inferCNV and transcriptional clustering. While widely accepted, these methods cannot fully replace genomic or histopathological validation, and the exact boundaries between malignant and non-malignant epithelial-like cells require further experimental confirmation.Fourth, pseudotime and trajectory inference analyses (Monocle and Slingshot) provide computational reconstructions of dynamic cellular processes rather than true temporal measurements. Therefore, the inferred differentiation trajectories should be interpreted as statistical models rather than definitive lineage relationships. Fifth, although CellChat analysis identified potential GRN-SORT1 and THBS1-CD47/CD36 signaling interactions between C3 *UBE2C*+ tumor cells and stromal/immune compartments, these interactions remain computational predictions and require validation through spatial transcriptomics and functional experiments. Finally, the *in vitro* validation was limited to two SCLC cell lines and focused on *UBE2C* knockdown. While these results support its oncogenic role, they cannot fully recapitulate the complexity and heterogeneity of SCLC tumors *in vivo*.

Currently, there are no clinically approved targeted therapies specifically against *UBE2C*. Overall, *UBE2C* is considered a promising but not yet clinically actionable therapeutic target. Therefore, further research is needed in the future to validate the findings of this study. By utilizing a variety of scientific research methods, we can further refine these findings and advance subsequent clinical translation and related drug development.

Overall, through its application in both basic and translational research, snRNA-seq technology has identified new cellular subpopulations and elucidated related cellular pathways, thereby laying the groundwork for the discovery of potential drug targets.

## 5 Conclusion

Overall, our study provided an integrative single-nucleus view of SCLC malignant cell heterogeneity and identified *UBE2C*+ proliferative tumor cells as a functionally validated malignant state connected to immune-stromal regulatory networks. These findings suggested that *UBE2C* may function as a functionally relevant regulator of malignant phenotypes in SCLC cells and identified GRN-SORT1 and THBS1-CD47/CD36 as candidate communication axes requiring further experimental validation. Although further validation is needed in larger cohorts and additional experimental models, this study nevertheless provides a framework for investigating the plasticity of tumor cells in small cell lung cancer and identifying potential regulatable targets.

## Data Availability

The original contributions presented in the study are included in the article/**Supplementary Material**. Further inquiries can be directed to the corresponding author/s.
