# Supplementary material for: Integrative single-nucleus transcriptomic analysis identifies UBE2C+ proliferative tumor cells and immune-stromal regulatory crosstalk in small cell lung cancer
Source: Front Immunol. 2026 Aug 4;17:1902738. doi: 10.3389/fimmu.2026.1902738 (PMC13481786; doi:10.3389/fimmu.2026.1902738)
Supplement: Supplementary file 1 [file Table1.docx]

| **Gene** | **Primer/siRNA** | **Sequence (5′→3′)** |
| --- | --- | --- |
| UBE2C | Forward primer | CTGCCTTCCCTGAATCAGACAACC |
| UBE2C | Reverse primer | GTGGTGGGCATACTTACCGTCTCC |
| GAPDH | Forward primer | GGAGCGAGATCCCTCCAAAAT |
| GAPDH | Reverse primer | GGCTGTTGTCATACTTCTCATGG |
| si-UBE2C-1 | Sense | GGAUUGAAGAAGUCUACAUTT |
| si-UBE2C-1 | Antisense | AUGUAGACUUCUUCAAUCCTT |
| si-UBE2C-2 | Sense | CCUGCAAGAAACCUACUAATT |
| si-UBE2C-2 | Antisense | UUAGUAGGUUUCUUGCAGGTT |
| si-Ctrl | Sense | UUCUCCGAACGUGUCACGUTT |
| si-Ctrl | Antisense | ACGUGACACGUUCGGAGAATT |
